# Identifying System-Level Strategies to Engage in HPV Prevention Across Oral Health and Primary Care Settings

**DOI:** 10.3390/vaccines12101194

**Published:** 2024-10-19

**Authors:** Sarah B. Maness, Kathleen L. Egan, Leslie Sanchez, Mahmoud Al-Dajani, Essie Torres, Andres Flores, Alice R. Richman

**Affiliations:** 1 Department of Health Education and Promotion, East Carolina University, Greenville, NC 27834, USA; osoriopascuall23@ecu.edu (L.S.); richmana@ecu.edu (A.R.R.); 2 Department of Implementation Science, Division of Public Health Sciences, Wake Forest University School of Medicine, Winston-Salem, NC 27101, USA; klegan@wakehealth.edu; 3 School of Dental Medicine, East Carolina University, Greenville, NC 27834, USA; aldajanim19@ecu.edu; 4 Office of the Vice Chancellor for Research, University of North Carolina at Chapel Hill, Chapel Hill, NC 27599, USA; essie.torres@unc.edu; 5 Department of Surgery, College of Medicine, University of Cincinnati, Cincinnati, OH 45267, USA; floresa9@ucmail.uc.edu

**Keywords:** human papillomavirus, cancer prevention, oral health, primary care

## Abstract

Introduction: HPV vaccination prevents most HPV-related cancers, yet uptake remains low. HPV is linked to an estimated 70% of oropharyngeal cancers (OPCs) in the US and outnumber cases of HPV-related cervical cancers. Not all OPCs can be detected through routine screening, making HPV vaccination a more effective primary prevention strategy. However, bridging primary and oral healthcare faces challenges due to a lack of referral networks between practices. The purpose of this study is to identify key infrastructure elements and policies, as well as HPV prevention strategies, among an academic practice network of dental clinics and partnering community health clinics in a southeastern state. Methods: Researchers held interviews with directors and focus groups with staff at six dental clinics and eight associated community clinics in a southeastern state. Interviews and focus groups at dental and community clinics were analyzed by two study team members using thematic analysis with Nvivo software. Results: A total of 90 participants participated in all focus groups and interviews (N = 14 interviews, 10 focus groups (5–13 participants per focus group). Most participants identified as white (58.9%) and female (70%), with an average age of 38.5 years. Researchers identified nine key study themes: three specific to the dental clinics’ HPV conversations with patients, two related to community clinics’ vaccine provision, and four involving the relationship between the dental and co-located community clinics. Dental clinic staff do not currently discuss HPV with patients. They are open to discussing HPV with patients but anticipate barriers that require preparation to overcome them. Community clinics have demonstrated previous success with HPV vaccination, but patients over the age of 18 face financial barriers to vaccination. Community clinics and dental clinics report that they do not currently have existing referral networks but are open to a referral system between practices if infrastructure is put into place to support it. Conclusions: Our findings indicate that there is interest in, and potential for, increased discussion of HPV with dental patients and collaboration between dental and community clinics for HPV vaccination referral. The results of this investigation can be used to develop intervention strategies to increase HPV vaccination through referrals between dental clinics and nearby community clinics. Ultimately, this work can reduce health inequities in HPV-related cancers, serve as a model for US dental practices, and possibly influence public health policy.

## 1. Introduction

Human papillomavirus (HPV) is the most common sexually transmitted infection (STI) in the United States (US) and is associated with genital warts and anogenital and oropharyngeal cancers [1,2]. There are over 200 HPV types, with at least 14 high-risk types [3]. Two strains of HPV (HPV 16 and HPV 18) are responsible for most HPV-related cancers [4,5]. While pap screening and HPV DNA testing can detect early cervical cancers, no standard screening tests exist for other types of HPV-related cancers, including oropharyngeal cancer [6]. HPV is linked to an estimated 70% of oropharyngeal cancers (OPCs) in the US and outnumber cases of HPV-related cervical cancers [7]. Men are more likely to be diagnosed with OPCs than women, and the incidence of OPCs is on the rise [8]. Not all OPCs can be detected through routine dental exams unless they are at a clinically advanced stage, making HPV vaccination a more effective primary prevention strategy [9]. The FDA has expanded indications of the HPV 9-valent vaccine to include oropharyngeal and other head and neck cancers [10].

Most sexually active people (>80%) will contract HPV during their lifetime [11]. Due to the pervasive nature of HPV infection, vaccination prior to exposure is a highly effective HPV prevention method [12]. The Advisory Committee on Immunization Practices (ACIP) recommends routine HPV vaccination at 11 or 12 years of age, although vaccines can be started at age 9. ACIP also recommends vaccination through age 26 if not previously vaccinated when younger. Adults aged 27–45 may be vaccinated through shared clinical decision making with their providers [13]. HPV vaccination prevents most HPV-related cancers [14], yet HPV vaccination among eligible populations in the US remains low [15]. Data from the 2022 National Health Interview Survey (NHIS) indicate that 38.6% of children aged 9–17 and 47.4% of young adults aged 18–26 received at least one dose of the HPV vaccine [15,16]. NIS-Teen data indicate that in 2022, HPV vaccine initiation among 13–17-year-olds did not increase for the first time since 2013 [17]. The 2019 NHIS data show that only 15.6% of adults aged 27–45 had ever received the HPV vaccine [18].

Many studies have shown that provider recommendation for HPV vaccination is positively associated with vaccine uptake [19]. Promoting the HPV vaccine in non-traditional locations such as dental offices is a strategy that could increase vaccine uptake. The American Dental Association has also encouraged dentists to educate themselves and their patients on HPV, recommend HPV vaccination, and screening for oral and oropharyngeal cancers [20].

Although recommendations now support dentists engaging in HPV education and prevention, data show that dentists are not well prepared to engage with patients about HPV [21]. Previous studies examining dental health provider knowledge, opinions, and the practice of HPV vaccine communication and HPV/oropharyngeal cancer prevention found that optimal knowledge of prevention and early detection was lacking [22,23,24]. Moreover, other studies that have specifically assessed HPV-related OPC knowledge and practice among dentists were found to be limited, and these providers do not focus on specific partnerships with medical practitioners [24,25,26]. Research using the National Survey of Children’s Health has indicated that, above the age of nine, children have more dentist visits than physician visits. This gives dentists an opportunity to address health in older children, including HPV [27].

The purpose of this study is to identify key infrastructure elements and policies, as well as HPV prevention strategies, among an academic practice network of dental clinics and partnering community health clinics in a southeastern state. This study is unique in that it examines the functioning of co-located community clinics and academic networks of dental clinics to see how dental health providers may play a role in not only HPV education but also in referring to community clinics for HPV vaccination. Bridging primary and oral healthcare for rural, low literacy, and medically underserved areas faces many challenges due to the lack of formal communication and referral networks between many practices [28]. The academic network of dental clinics and co-located community clinics in the current study are strategically located throughout a southeastern US state in medically underserved, primarily rural areas, which allowed us to identify implementation strategies that may be most effective in this context. This study will also lay the groundwork for developing a model to increase HPV vaccination among unvaccinated populations served by co-located medical and dental centers.

## 2. Methods

### 2.1. Participants and Setting

Researchers interviewed 8 dental clinic directors and held 5 in-person focus groups (6–13 participants per focus group) with staff from university-associated dental clinics in a southeastern state. Researchers also interviewed 6 clinic directors and held 5 in-person focus groups (5–6 participants per focus group) with staff at nearby community clinics. All university-associated dental clinics (n = 8) were strategically placed in medically underserved areas across [the state in which the study was conducted], and all but one are located in a rural county.

Phase I of the study involved one-on-one phone interviews with the Clinic Directors or over Microsoft Teams or Webex [29,30]. The interviews lasted approximately 30 min, and the participants received a USD 25 gift card as compensation.

The second phase of the study included focus group discussions, which comprised clinic providers, directors, and staff at the same clinics in which clinic directors participated in interviews. Participants received refreshments during the one-hour focus group. Topical guides for the interviews and focus groups were developed to measure office policies, current HPV prevention strategies, perceived facilitators and barriers to referring patients across practices, identifying gaps and opportunities for HPV prevention, and ideas to bridge the medical and dental divide for practical prevention efforts.

### 2.2. Measures

#### 2.2.1. Interview Guide

**Figure 1:**
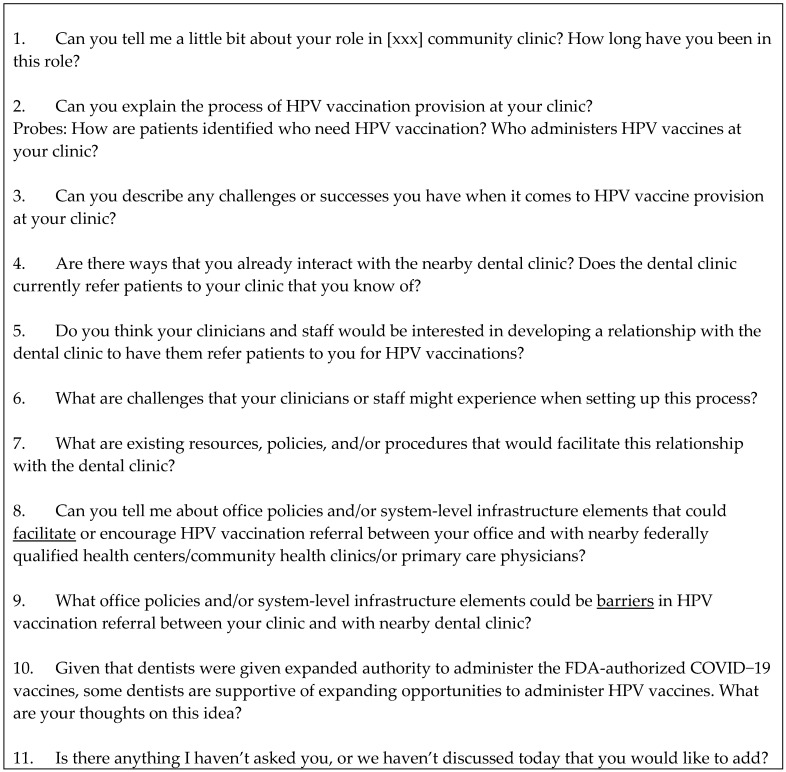
Sample interview guide.

The interview guide for community clinic directors consisted of 11 content questions. A sample interview guide can be seen in Figure 1. The interviews opened with asking participants about their role in the clinic, followed by explaining the process of HPV vaccine provision, including successes and challenges. The interview then focused on existing interactions with the nearby dental clinic, interest in developing a relationship for referrals, and challenges, resources, or policies that could accompany referrals for HPV vaccination between clinics. Lastly, participants were asked their thoughts about dentists administering HPV vaccines.

Although similar to the interview guide for community clinic directors, the interview guide for dental clinic directors contained several key differences. This interview guide opened with a discussion of directors’ roles in the clinic but then moved into questions about current oral cancer screening strategies implemented in their day-to-day clinical practice. The next section of questions focused on HPV education, how staff might engage in it, whether staff would be interested in this education, potential challenges, facilitators, and existing resources and policies. The interview concluded by asking about interactions with nearby community clinics, challenges, facilitators, or policies that could accompany referrals for HPV vaccination between clinics. Participants were also asked their thoughts about dentists administering HPV vaccines. All interviews were finalized with open-ended questions, recording additional thoughts before completing the discussion.

#### 2.2.2. Focus Group Guide

**Figure 2:**
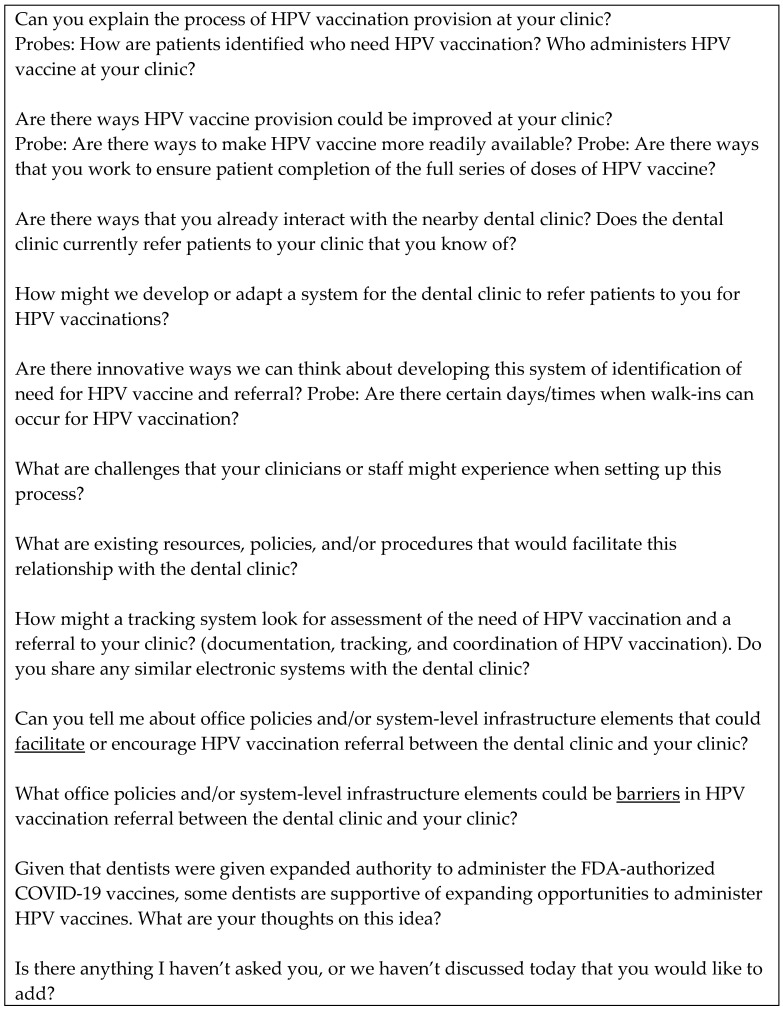
Sample focus group guide.

Community clinic focus groups used a topical guide that began with background information (set up, greeting, roles, informed consent, purpose, how to participate, ground rules, introduction, and icebreaker). Next, two content questions asked about the provision of the HPV vaccine and how it could be improved. The rest of the questions were related to bridging primary care and oral health care for patients with HPV vaccination. Participants were asked if they already interacted with nearby university-associated dental clinics, how referral systems could be set up, and resources, challenges, and facilitators for referrals between clinics. Before closing, participants were asked their opinion about dentists administering HPV vaccines and if they had any additional thoughts about discussion topics. A sample of the instrument utilized to guide the discussion can be found in Figure 2.

The topical guide for the dental clinic focus group began with a set up, a greeting, informed consent, instructions on how to participate, and the setting of ground rules. Following introductions and an icebreaker, participants had a conversation surrounding 10 content questions. The first question asked about how staff may engage in HPV education and vaccine promotion, followed by asking about challenges and what would make it easier to have these conversations and build them into routine practice. Additional questions were asked about relationships and referrals with community clinics, what a tracking system might look like, and policies that could facilitate or become barriers to HPV vaccine referrals between offices. The focus group ended with a discussion about thoughts on dentists administering HPV vaccination and the opportunity to discuss anything that was not previously asked.

#### 2.2.3. Demographic Survey

Focus group participants completed a five-question demographic survey prior to the beginning of the focus group session. Questions included job/profession, years in their job/profession, sex, race/ethnicity, and age.

### 2.3. Qualitative Analyses

Interviews and focus groups were audio-recorded and transcribed verbatim. Transcripts were checked for accuracy by study staff. Interviews and focus groups at dental clinics and community clinics were analyzed together. Two study team members read all transcripts and developed a codebook. The team used Nvivo software to code all transcripts. One researcher read and coded all transcripts, and a second coder analyzed 30% of the transcripts to confirm the reliability of the data. These coders met repeatedly to go over discrepancies and review findings. Codes were reviewed by location (community clinic or dental clinic) and method (interview or focus group). We then used thematic analysis to determine key themes from the interviews and codebooks. All study procedures received approval by the [blinded for review] Institutional Review Board.

## 3. Results

A total of 90 participants participated in all focus groups and interviews. Most participants identified as white (58.9%) and female (70%), with an average age of 38.5. Overall, community clinic interviews and focus group participants (n = 32) were primarily nurses (71.9%) with more than nine years in the profession (37.5%). Among dental clinic directors and staff (n = 58), participants were general dentists (31%), dental assistants (29.3%), and dental students (22.4%). These participants were split between those who were less than two years into their profession (24.1%) and those who were more than nine years into their profession (31%).

Researchers identified nine key study themes: three specific to dental clinics’ HPV conversations with patients, two related to community clinics’ vaccine provision, and four involving the relationship between each type of clinic. Qualitative findings are consistent across clinic director interviews and staff focus groups unless otherwise noted. The themes are discussed in detail below (Table 1).

### 3.1. A Few HPV Discussions with Patients at Dental Clinics

Dental clinic staff reported that although they screened all new patients for oral cancer and at each periodic examination, they largely do not discuss HPV with their dental patients, even during oral cancer screenings. However, directors and staff are open to these conversations occurring and providing HPV information to patients.

  “*So, like, definitely oral cancer is a screening done at every hygiene appointment. But HPV is and it’s something that I personally never had a discussion with about with patients before. But as you’re reading that, I was thinking it would be easy to implement when you’re doing the oral cancer screening.*”

P1, Dental Clinic Focus Group 3

### 3.2. Dental Clinic Staff Perceive Barriers to HPV Conversations with Patients

Despite interest in having conversations about HPV with patients, dental clinic staff identified barriers to having these conversations. Barriers identified included not having enough time, HPV being a stigmatized topic, and vaccine hesitancy. Staff mentioned that schedules are full and there are many tasks during narrow appointment times. They do not have enough time to perform all the counseling that they want to carry out. Clinics also do not have dialogs or plans to guide HPV conversations with patients; however, many staff shared ideas for overcoming these barriers.

  *I mean, our schedules usually are pretty well filled, and we’re challenged to get it done and get done quickly. And when go through health histories and you go through medical histories and medication histories and then you’ve got to work through, “I got to get this patient, No, I got to take care of this too”. I’m kind of taking care of this procedure sometimes the short sight is not as good as the long sight, or the long sight is not as good as the short sight. So, time can be a big constraint*.

P1, Dental Clinic Focus Group 5

Staff and directors often mentioned stigma as a barrier to HPV conversations with patients because HPV is sex-related and seen as taboo. This concern was especially common when considering conversations with patients who are underage and have a parent present. Staff are unsure what is appropriate to discuss with the patient and feel uncomfortable not knowing what may cause offense. Directors feel that HPV conversations could be uncomfortable for both patients and staff, especially when patients are under 18 and their parent is present.

  *And what age are you pertaining this to a child and their parent? I mean, to me, I feel like I would run across someone in our community that would cause a fit- thinking that their child was sexually active or at that stage in life or something. I don’t really know where you would draw that line at. It like I feel like that as a provider health provider, discussion, and conversation. I don’t feel very comfortable talking about that.”*

P4, Dental Clinic Focus Group 4

  *Yeah, I mean, me personally, what I think I haven’t seen any studies on it or looked at it, but I’m thinking there someone may be uncomfortable talking about anything this sex related. And I know we’re in a really you know, we’re in a charged environment now where, you know, whether you’re talking about gender or sex or anything else, someone’s behaviors, whether you’re being too judgmental or whether you’re, you know, offending someone, people are offended easily.*

Dental Clinic Interview 2

A third barrier to HPV-related conversations with patients is vaccine hesitancy. Staff mentioned that HPV is not only a sensitive topic because it is sexually transmitted but also because of anti-vaccine sentiments. Staff and directors are concerned about the lack of trust in the medical establishment and vaccines, especially since the COVID-19 pandemic.

  *First off, I think it could be a sensitive topic on the patient end as well as the staff end. Because it’s typically, not always, typically correlated with sexual activity of one way or another, and in the age group that they want to vaccinate, patients may be very reluctant to spill information to you, certainly in the presence of a parent. So, discussing it with that age group. And then certainly right now people are vaccine resistant. They are vaccine reluctant. All the COVID stuff. So, they may not want to hear anything else about another vaccine. So that would be a challenge for sure.*

Dental Clinic Interview 6

Directors specifically mentioned in interviews that they do not have a dialog for staff and do not connect HPV with oral cancers. Most staff are not undertaking HPV education but have ideas about how it could be carried out using brochures, talking about it during oral cancer exams, putting vaccine questions on medical history, or having hygienists bring it up. Staff may feel uncomfortable or not knowledgeable enough to bring it up.

  *Yeah. Okay. Currently, I don’t think we’re connecting the HPVs with oral cancers and that’s probably been the lack of knowledge or the lack of experience on staff. And now how might we do that going forward? That’s a good question there. I think, and the hygienist also recommended, putting a single question into our medical history to indicate whether they have or have not had the HPV vaccine and then whether or not they would want that going forward.*

Dental Clinic Interview 6

### 3.3. Dental Clinic Staff Request Preparation for HPV Conversations with Patients

To move forward with HPV conversations during dental care visits, staff requested preparation in the form of infrastructure support and training. Ideas for preparation included brochures, incorporating a medical history question, and staff training. In nearly every staff focus group, a participant brought up the idea of using brochures to assist with conversations about HPV with patients. Brochures were mentioned as both something that could help the patient feel that they could bring the topic up to the provider if seen in a waiting room or something that could assist with the issue of time if given to the patient by the provider during an appointment. Brochures could also give patients the next steps regarding where to go if they want to learn more or receive HPV vaccination. Hygienists routinely give patients goodie bags to take home, and the brochures could be easily placed inside.

  *You know, it’s like, what do you think about a brochure or something like that? Somebody could hand somebody if you can’t have this big discussion right there in the chair, because usually people are here to get something done, but it has to be done within a finite amount of time. It’s not a real open ended appointment. You can’t bring them in next week for another discussion or something like that.*

Dental Clinic Focus Group 2

Another common idea for assistance with HPV conversations was to put a question about HPV into the medical history form. This would ensure that every patient is asked about HPV in a routine way, along with other aspects of their medical history. Staff noted that medical histories include more than just dental information, including social, employment, and family history, making it an appropriate place to ask about HPV.

  *We do every patient that comes into the office. We have a very thorough like medical history form that has like their medical histories, but also like the social part, like even employment history. If that was a question that was added on there, then we verbally go over with the patient, the medical history, like they don’t just fill it out and we look at it like it’s actually a conversation that we have with every patient.*

Dental Clinic Focus Group 3

In addition to infrastructure support to ease bringing up the topic of HPV, staff, and directors want education and training on the topic. Dental clinic staff asked many questions about HPV and HPV vaccination (age, eligibility, cost, how is it transmitted, whether vaccination is required, side effects, who can receive it, why it is not available for over 45s, etc.) and often directly stated not knowing much about HPV. Directors want training, practice, and communication skill-building for their staff.

  *I might would feel more comfortable discussing it honestly if I was more educated.*

Dental Clinic Focus Group 2

  *Yeah, I think, I think some sort of education, I mean, of my long-standing assistant, every one of them has worked with me with a cancer center patient before. And then they’ve gotten very you know, it’s not something dental assistants usually see. But some of these some of these have been pretty ugly. Pretty ugly. I think I think they’ll be comfortable doing it within the bounds that I just discussed as long as they’re educated a little bit.*

Dental Clinic Interview 3

### 3.4. Community Clinics Have Established HPV Vaccination Provision Processes

Community clinics have established processes for vaccine provision and have demonstrated success with HPV vaccination. Community clinics offer HPV vaccination to eligible patients, either by checking the [state in which the study was conducted], the Immunization Registry for their eligibility, or through electronic health records (EHRs). One location offers vaccination to all in the Family Planning and STD clinic. It is overwhelmingly nurses who administer vaccines, with two locations mentioning doctors or medical assistants. About half report walk-ins for vaccines; others state they do not turn people away but do not advertise walk-ins. Directors each described how patients are identified and offered the HPV vaccination, and all described a set process based on age guidelines or automated notices that come up in a patient’s chart. Some offices refer to immunization clinics, while others offer the vaccine.

  *Well, we follow the recommendations for all of our patients, every child or, you know, anybody in the age range to receive HPV. When- we check the [state in which the study was conducted] immunization registry to see who’s eligible and then offer that to everyone who is eligible for it.*

Community Clinic Interview 1

  *So, for our, um our child health clinic, the young adolescence when they turn 11. When we do their TDap and their meningitis vaccine we offer HPV vaccine, so we try to really encourage the parents to, uh, to get that and most of them are and most of them will take the vaccine for their kid.*

Community Clinic Interview 4

Staff and directors often mentioned success in providing the HPV vaccination, quoting high vaccine completion rates, rapport, and relationships with patients. Community support, such as vaccine cost assistance, federally subsidized pharmacy, the VFC program, and transportation assist support with these successes, as well as clinic processes such as mail reminders, scheduling, print-outs, and patient education. Two locations specifically mentioned doing well in coverage rates in recent years.

  *And one of the things that we looked at was actually our HPV vaccine rates through our BFC program. And we’re actually doing very well in our statistics. We- I think- had a completion rate of 72% is what we had, which was one of the best.*

Community Clinic Focus Group 1

  *And I think we would have success because our patients, most of our patients have been coming to us for years and they trust us, and they know us. You know, when they see us out. We have a good rapport with people.*

Community Clinic Interview 2

### 3.5. Community Clinic Patients Face Financial Barriers to HPV Vaccination

A wide variety of challenges to HPV vaccination were mentioned across community clinic locations. The most frequently mentioned challenges were that the vaccine is cost-prohibitive if over 18 or due to insurance barriers such as the need for proof of income, followed by a limited volume of eligible patients, administrative issues, no-shows for appointments, and resistance from parents.

The financial barriers for patients to receive HPV vaccination provision at community clinics are well noted by both staff and clinic directors. The main issue is that without insurance, HPV vaccination is expensive. Children can be covered through the Vaccines for Children program, but adults wanting vaccination often face issues with payment. In addition, patients on Medicaid need to get vaccinated by their primary care physician, or their insurance will not cover it.

  *All of our patients are uninsured. So, it is quite an eyeopener for us. So, 250 for a shot and it’s a three shot series*

P5 Community Clinic Focus Group 2

  *Now adults they would have to pay for it, right? For the HPV then if their children they would be free any of them that they wanted to refer to us. We’d be happy to give. Yeah, because it’s what a hundred and how much is it…*

P3 Community Clinic Focus Group 5

  *The vaccine isn’t even given on a sliding scale. It is very, very expensive. So, it’s $254. And then they charge a $20 administration fee. So, it’s $274 a dose, for this. If they don’t have insurance or if they’re too old to qualify for VFC to get the vaccine. So, is that something you guys were even aware of? How expensive it is?*

Community Clinic Interview 2

### 3.6. Community Clinics and Dental Clinics Do Not Report Close Relationships

Community clinics and dental clinics largely do not have very close relationships, although they are mostly aware of each other and sometimes refer patients between practices. When asking community clinic staff and directors about referring to dental clinics, there were often questions and uncertainty about the details of these referrals. Multiple community clinic directors stated that there were no set referral policies for dental clinics. Standing orders would facilitate a relationship.

  *We have taken stuff to them. Like do they know? I mean, they obviously they kind of know we exist. But you’re rotating through with it being, you know, I guess what fellows? Or whatever. They’re rotating through-*

P2 Community Clinic Focus Group 1

Dental staff and directors were more likely to be aware of the possibility of referring patients to community clinics. Staff mentioned telling patients to go to a community clinic if they did not have regular care, yet sometimes referrals are made to other places, such as the health department. Dental clinics generally only refer to oral surgeons, but if other medical-related issues come up, then they perform a consultation with a primary care provider (PCP). Staff reported it is also common to perform consultations for high blood pressure or A1Cs before clearing for dental treatment. There is also some word of mouth or encouragement to obtain a PCP and letting patients know where to go if they do not have one, such as to the health department or dental clinics. Staff spoke predominantly about oral surgeons and PCP medical consultants, while directors often mentioned referring to the health department or PCPs.

  *But yeah, we have their pamphlets, so we send people to the community care clinic a lot.*

P5, Dental Clinic Focus Group 4

  *We don’t have the wonderful relationship that many of our [dental clinic]’s do with or co-located FHQ. For a variety of reasons, however, other clinics that we referred to and PCP’s piece that we do that, you know, regularly. Well and we do refer to them, but it’s a, it’s a difficult situation.*

Dental Clinic Interview 8

### 3.7. Community Clinics and Dental Clinics Are Open to Shared HPV Vaccination Referrals

Community clinics and dental clinics are open to relationships for HPV vaccine referrals. There is a desire for collaboration under formal agreements and for the mutual good of patients. Multiple ideas for methods of referrals include requesting appointments via a website, dental clinic calling, and giving a card for patients to call. Dental clinics do not currently track whether someone has had the HPV vaccine. A few ideas for how to do so were to add to the EHR, including putting in notes or writing a consultation that they are interested in the vaccine. Many mention wanting a way to follow up to see whether the patient went to the appointment.

  *If it happens, we would probably do like an MOU just like a mutual understanding. So just so one can’t say no, we didn’t say we did that, or we didn’t say we’d do that. You know we would want both parties to agree upon how we’re going to make it work. I mean we do that with the schools.*

P1, Community Clinic Focus Group 5

  *Yes. We always try to collaborate and like I always call it playing nice in the sandbox. So, we try to always play nice in the sandbox because unless we all work together, we’re not going to be able to provide the things that people need, you know, that they can’t afford. That’s right.*

Community Clinic Interview 2

  *I would think so. I mean, of course, you know, we want everyone to get vaccinated. So, I think obviously if you have patients that need that vaccination. And then the other thing to a lot of those patients or some of those patients may not have a provider that they regularly see, so we may be able to speak with them and just offer our services until them you know, these are the things that our clinic offers.*

Community Clinic Interview 3

### 3.8. Infrastructure Is Needed for HPV Vaccine Referrals between Community Clinics and Dental Clinics

Although there is interest in referring patients for HPV vaccination, infrastructure is needed because there are no shared charts or referral systems. Community clinic and dental clinic staff appeared not to know what systems the other used.

  *I don’t know what they use, but we use epic here.*

P1 Community Clinic Focus Group 4

One staff member described how, currently, they could write a consultation and send it over but that there are no shared systems. Not all offices use the same electronic medical records, and it is not clear which clinics have access to resources such as being a member of the [state in which the study was conducted] Health Exchange Information Authority or having access to the [state in which the study was conducted] Immunization Registry. Dental clinics have their own database that is not shared outside of the associated dental school.

  *So yes. And so, and that’s where like I guess NCLR would be helpful. But again, getting the systems- not every office has EPIC. They’re all different. And if they’re not communicating with NCIR, then you don’t know that they haven’t had that shot. You know? Or you don’t know if they’ve had one. Did you get the other one? “It’s undocumented”.*

P2, Community Clinic Focus Group 1

  *Not unless they, I don’t know what the dental clinic has and are they a member of [state in which study was conducted] health exchange information authority?*

P1, Community Clinic Focus Group 5

Policies that could facilitate HPV vaccination referrals include a medical consult form, communication between clinics, and standing orders for vaccines. There is a need to streamline infrastructure. For example, having forms completed ahead of time for billing and proof of income, understanding vaccine availability and appointment times, and knowing what the cost will be ahead of time. Dental clinic and community clinic directors find that there should not be many barriers to collaboration, but there is a need to establish the process. Transportation was also mentioned as a barrier as sometimes the other clinic is not close.

### 3.9. Community Clinic Staff Support Dentists Giving HPV Vaccination, but Dentists Have Concerns

Community clinic staff largely supported dentists giving HPV vaccination. Thoughts on this included that dentists could give other shots, so if they have the training and can figure out logistics, it is a good idea. Dentists were already giving the COVID-19 vaccine, so community clinic staff did not see HPV vaccination as a big leap.

  *What’s the point of referring someone out when you could just give it yourself? I mean, you have a medical degree of dentistry. But like he said, they’re stickin’ gums and medicine in my mouth, so I think they can stick a vaccine in my arm.*

P4, Community Clinic Focus Group 2

  *Basically, just like my that if they can inject my mouth and numb me in just the right way, I think they’re perfectly skilled enough to do that.*

P5, Community Clinic Focus Group 3

  *I mean, one less thing we got to do.*

P4, Community Clinic Focus Group 4

Dental clinic staff, many of whom are dentists, were much less eager to support dentists giving the HPV vaccination. Some dentists stated that they did not personally want to perform it but would be okay with other dentists performing it. The main concerns are that it is outside the scope of a dentist’s practice, that they do not have enough time, and liability.

  *When I think of vaccines, I think of a pharmacist or a doctor giving them. And I’m not saying we’re not doctors, but we’re dentists, and giving a shot not in the mouth just seems abnormal to me.*

P3, Dental Clinic Focus Group 1

  *It is added work, also there is liability with it. I’m not sure even the dentist can give the vaccine. There might be. I know you need some certain certificate to give the vaccine, but I don’t think the dentists do it.*

Dental Clinic Focus Group 4

HPV vaccination is seen as a burden on dental practices already stretched for time and staffing. Reasons for not personally wanting to perform the vaccination include: it takes more time, they are understaffed, it is a liability, and it requires more money for refrigeration and handling the billing processes. Some just feel it should be left to other medical professions.

  *I think in an educational setting, it’s not a good idea. I just don’t think there’s extra time involved. I have no open appointments. I have no extra assistance. Matter of fact, I’m short of assistance. So, the burden put that additional burden on me, is going to cut down the ability of me to be self-sustaining.*

Dental Clinic Interview 5

It is also a common idea among dental clinic staff that if it was necessary, like during the COVID-19 pandemic, they would give vaccines, but they live in areas with plenty of locations to obtain vaccines, and they do not see a need to add it to what a dentist does. A few dentists mentioned they would perform the vaccination with no concerns, were interested, or would want more training.

## 4. Conclusions

Our findings indicate that there is interest and potential for increased discussion of HPV with patients and collaboration between community clinics and dental clinics for HPV vaccination referral. However, there are multiple barriers to overcome for dental staff to feel comfortable talking about HPV with patients, infrastructure to put in place, and the need to formalize a collaboration with community clinics.

Results are consistent with the literature, showing that limited time and not knowing enough about HPV are reported barriers for dentists and dental hygienists providing HPV education [31,32]. A recent qualitative study conducted among dentists and hygienists in the midwestern United States reported similar findings to our study, including that staff were open to recommending HPV vaccination but needed more education on how to undertake this. This study found that only one participant had ever discussed HPV vaccination with a patient, and the main themes included a lack of self-efficacy to promote vaccination, lack of knowledge of HPV and the HPV vaccine, and fear of negative consequences (patient reactions) from promoting the vaccine [32].

Dental clinic staff reported feeling uncertain and uncomfortable about having conversations about HPV with patients under the age of 18 with their parents present. Previous research indicates that parents feel dentists are qualified to counsel about HPV and HPV vaccination and are comfortable receiving HPV information from them [33]. It may be helpful to include this information in training for dental clinic staff in preparation for having HPV discussions with patients.

Our findings that dental clinic staff are open to providing HPV education and referring to other providers for HPV vaccination but not administrating the vaccine themselves, aligns with previous research [26,34]. Including HPV vaccination in the scope of dental practice is a topic that has been increasingly raised in recent years, yet the provision of vaccines by dentists remains low [35,36,37].

We found that community clinics have established processes for vaccine provision, which indicates that they would be able to facilitate vaccination if sent referrals from dental clinics. A large barrier mentioned for adult patients is the high cost of the HPV vaccine. Many practices see patients who are uninsured or underinsured. Youths under the age of 18 who are Medicaid-eligible or underinsured can be covered under the Vaccines for Children program [38]. As a recommended vaccine, the HPV vaccine can also generally be covered by private health insurance plans up to the age of 26. Patients over the age of recommended vaccination may still be eligible for vaccination (age 27–45) but face a high cost [39]. Without insurance coverage, the list price of the HPV vaccination can be several hundred dollars, and the Merck list price for GARDASIL 9 as of October 2024 is USD 286.78 [38,40]. Research among young adults indicates that even among insured adults, the cost is perceived as a significant barrier to getting vaccinated [41]. If a new referral process is set up, yet patients perceive they cannot afford the vaccine, the collaboration will not be successful. As of 2022, 43 states provided state Medicaid coverage for HPV vaccination in adults up to age 45, including the state in which the current study was conducted [42]. There are also vaccine assistance programs for underinsured adults [43]. In a future collaborative referral program, assisting patients in determining what they will pay for the vaccine may be key in facilitating vaccine uptake.

The findings that community clinics and dental clinics do not have close relationships or formal processes for referrals present a challenge in creating infrastructure to facilitate a formal referral process for HPV vaccination. There is a need to identify what medical record systems each practice uses and if they are complementary. There may be ways to coordinate within existing practices or to determine if a unique system will need to be created to refer patients between practices. Physicians have previously identified a lack of adequate dental referral systems as an issue that a shared app or electronic system could ameliorate [44]. If dental offices have different electronic health record systems, it may be impossible to bridge the gap using existing software. However, there are possibilities for developing an alternate referral strategy that tracks patient appointments and vaccine uptake outside of the electronic health record system. This would improve current practices found in our study where offices did not know if patients followed up on a referral. Our findings are also relevant in that dentists did not report feeling comfortable giving the HPV vaccine themselves. This indicates the need for this innovative intervention to increase HPV vaccination utilizing referrals from dental settings.

This work is not without limitations. All data are self-reported, which may include potential bias. In some locations, clinic directors participated in both director interviews and focus groups, and we were unable to distinguish where viewpoints came from the same participant. We conducted this study within an academic practice network of dental clinics, and the findings may differ in private dental clinics. In addition, the demographic survey was given before focus groups, but not interviews. Future interventions should take into consideration the multiple barriers identified in this study.

We show the feasibility and acceptability of creating a referral system between academic practice networks of dental clinics and co-occurring community clinics serving medically underserved and rural areas. Clinic staff were amenable to the partnership but had practical ideas about what would need to be implemented for it to be successful. The practical next steps in beginning referrals for HPV vaccination between community clinics and dental clinics are to establish infrastructure for a referral process and shared electronic medical records and implement an HPV training module for dental clinic staff. This could involve developing and testing an intervention to educate dental clinic staff, implementing the intervention to refer to nearby clinics, and then evaluating acceptability and effectiveness in increasing vaccine uptake. It is also prudent to strengthen the relationships between each clinic, building the capacity for working together. This could begin with shared meetings between clinic directors or events that link staff from each co-located community clinic and dental clinic. The results of this research can be used to develop an HPV vaccine recommendation and referral action plan where dental clinics navigate patients toward primary care or local community clinics. Ultimately, this work can reduce health inequities in HPV-related cancers and serve as a model for US dental practices.

## Figures and Tables

**Table 1:** Summary of themes.

Theme	Description	Example Quote
Few HPV Discussions with Patients at Dental Clinics	Dental clinic staff screen for oral cancer but do not discuss HPV with patients, even during oral cancer screenings Open to starting these conversations	“*So, like, definitely oral cancer is a screening done at every hygiene appointment. But HPV is and it’s something that I personally never had a discussion with about with patients before. But as you’re reading that, I was thinking it would be easy to implement when you’re doing the oral cancer screening*.” P1, Dental Clinic Focus Group 3
Dental Clinic Staff Perceive Barriers to HPV Conversations with Patients	Barriers include time, stigma, and vaccine hesitancy	*And what age are you pertaining this to a child and their parent? I mean, to me, I feel like I would run across someone in our community that would cause a fit- thinking that their child was sexually active or at that stage in life or something. I don’t really know where you would draw that line at. It like I feel like that as a provider, health provider, discussion, and conversation. I don’t feel very comfortable talking about that*. P4, Dental Clinic Focus Group 4.
Dental Clinic Staff Request Preparation for HPV Conversations with Patients	Dental staff want HPV education: brochures, questions in medical history, and training	*I might would feel more comfortable discussing it honestly if I was more educated.* Dental Clinic Focus Group 2
Community Clinics have Established HPV Vaccination Provision Processes	Community clinics offer HPV vaccine to eligible patients and report successful efforts	*And one of the things that we looked at was actually our HPV vaccine rates through our BFC program. And we’re actually doing very well in our statistics. We- I think- had a completion rate of 72% is what we had, which was one of the best.* Community Clinic Focus Group 1
Community Clinic Patients Face Financial Barriers to HPV Vaccination	Community clinic challenges include the cost of vaccine for adults, insurance barriers, limited volume of eligible patients, administrative issues, no shows and resistance from parents	*All of our patients are uninsured. So, it is quite an eyeopener for us. So, 250 for a shot and it’s a three shot series* P5 Community Clinic Focus Group 2
Community Clinics and Dental Clinics Do Not Report Close Relationships	Co-located community and dental clinics usually know about each other and sometimes refer patients but often do not have clear policies or knowledge of this process	*We have taken stuff to them. Like do they know? I mean, they obviously they kind of know we exist. But you’re rotating through with it being, you know, I guess what fellows? Or whatever. They’re rotating through-* P2 Community Clinic Focus Group 1
Community Clinics and Dental Clinics are Open to Shared HPV Vaccination Referrals	Community clinics and dental clinics would like to work together for HPV vaccine referrals after establishing formal agreements. They would like to be able to follow up to see if the patient was vaccinated	*If it happens, we would probably do like an MOU just like a mutual understanding. So just so one can’t say no, we didn’t say we did that, or we didn’t say we’d do that. You know we would want both parties to agree upon how we’re going to make it work. I mean we do that with the schools. P1,* Community Clinic Focus Group 5
Infrastructure is Needed for HPV Vaccine Referrals Between Community Clinics and Dental Clinics	Clinics are not aware of what electronic health systems each other use or if they would be compatible for shared referrals	*So yes. And so, and that’s where like I guess NCLR would be helpful. But again, getting the systems- not every office has EPIC. They’re all different. And if they’re not communicating with NCIR, then you don’t know that they haven’t had that shot. You know? Or you don’t know if they’ve had one. Did you get the other one? “It’s undocumented”.* P2, Community Clinic Focus Group 1
Community Clinic Staff Support Dentists Giving HPV vaccination, Dentists Have Concerns	Community clinic staff are supportive of dentists to give HPV vaccine; they have the training and can give other shots. Dentists feel that barriers are outside their scope of practice, time, and liability	*I think in an educational setting, it’s not a good idea. I just don’t think there’s extra time involved. I have no open appointments. I have no extra assistance. Matter of fact, I’m short of assistance. So, the burden put that additional burden on me, is going to cut down the ability of me to be self-sustaining.* Dental Clinic Interview 5

## Data Availability

The participants of this study did not give written consent for their data to be shared publicly, so due to the sensitive nature of the research, supporting data is not available.
